# Determinants and predictors for the long-term disease burden of intracranial meningioma patients

**DOI:** 10.1007/s11060-020-03650-1

**Published:** 2020-10-19

**Authors:** Amir H. Zamanipoor Najafabadi, Pim B. van der Meer, Florien W. Boele, Martin J. B. Taphoorn, Martin Klein, Saskia M. Peerdeman, Wouter R. van Furth, Linda Dirven, on behalf of the Dutch Meningioma Consortium, Florien W. Boele, Linda Dirven, Wouter R. van Furth, Martin Klein, Johan Koekkoek, Frank Lagerwaard, Pim B. van der Meer, Saskia M. Peerdeman, Martin J. B. Taphoorn, Amir H. Zamanipoor Najafabadi, Wouter A. Moojen, Jaap C. Reijneveld

**Affiliations:** 1 Department of Neurosurgery, University Neurosurgical Center Holland, Leiden University Medical Center, Albinusdreef 2, Postal Zone J11-R, 2333ZA Leiden, The Netherlands; 2 Haaglanden Medical Center & Haga Teaching Hospitals, The Hague, The Netherlands; 3 Department of Neurology, Leiden University Medical Center, Albinusdreef 2, Postal Zone J11-R, 2333ZA Leiden, The Netherlands; 4 Leeds Institute of Medical Research at St James’s, St James’s University Hospital, Leeds, LS9 7TF UK; 5 Faculty of Medicine and Health, Leeds Institute of Health Sciences, University of Leeds, Leeds, LS2 9JT UK; 6 Department of Neurosurgery, Amsterdam UMC, Vrije Universiteit Amsterdam, Amsterdam, The Netherlands; 7 Department of Neurology, Haaglanden Medical Center, The Hague, The Netherlands; 8 Department of Medical Psychology, Brain Tumor Center Amsterdam, Amsterdam UMC, Vrije Universiteit Amsterdam, Amsterdam, The Netherlands

**Keywords:** Meningioma, Health-related quality of life, Neurocognitive functioning, Predictors, Determinants, Risk factors

## Abstract

**Introduction:**

Meningioma is a heterogeneous disease and patients may suffer from long-term tumor- and treatment-related sequelae. To help identify patients at risk for these late effects, we first assessed variables associated with impaired long-term health-related quality of life (HRQoL) and impaired neurocognitive function on group level (i.e. determinants). Next, prediction models were developed to predict the risk for long-term neurocognitive or HRQoL impairment on individual patient-level.

**Methods:**

Secondary data analysis of a cross-sectional multicenter study with intracranial WHO grade I/II meningioma patients, in which HRQoL (Short-Form 36) and neurocognitive functioning (standardized test battery) were assessed. Multivariable regression models were used to assess determinants for these outcomes corrected for confounders, and to build prediction models, evaluated with C-statistics.

**Results:**

Data from 190 patients were analyzed (median 9 years after intervention). Main determinants for poor HRQoL or impaired neurocognitive function were patients’ sociodemographic characteristics, surgical complications, reoperation, radiotherapy, presence of edema, and a larger tumor diameter on last MRI. Prediction models with a moderate/good ability to discriminate between individual patients with and without impaired HRQoL (C-statistic 0.73, 95% CI 0.65 to 0.81) and neurocognitive function (C-statistic 0.78, 95%CI 0.70 to 0.85) were built. Not all predictors (e.g. tumor location) within these models were also determinants.

**Conclusions:**

The identified determinants help clinicians to better understand long-term meningioma disease burden. Prediction models can help early identification of individual patients at risk for long-term neurocognitive or HRQoL impairment, facilitating tailored provision of information and allocation of scarce supportive care services to those most likely to benefit.

**Electronic supplementary material:**

The online version of this article (10.1007/s11060-020-03650-1) contains supplementary material, which is available to authorized users.

## Introduction

Although over 95% of meningioma patients have a non-malignant WHO grade I or II tumor [1], these patients still suffer from a clinically relevant disease burden, even after tumor resection, which can persist over time [2–6]. Compared with controls, meningioma patients report on average worse health-related quality of life (HRQoL) up to 9 years after surgery [3, 4]. Approximately 40% of patients have neurocognitive impairments, although these impairments are often not considered clinically meaningful [5–7]. However, not all meningioma patients have poor outcomes and it is currently unclear which factors are related to the long-term disease burden, while it might help to better understand the disease burden in meningioma patients. In the clinical setting, early identification of patients at high risk for a long-term disease burden facilitates timely provision of information and rehabilitation, and allocation of scarce supportive care services to those most likely to obtain benefit.

A limited number of published studies have reported a variety of variables associated with increased meningioma disease burden in the first years after treatment, primarily focusing on sociodemographic (e.g. higher age and lower educational level), tumor (e.g. larger tumor diameter and higher WHO grade) and treatment characteristics (e.g. higher Simpson grade and receiving radiotherapy) [3, 7]. However, there are no published studies on the possible factors associated with the long-term disease burden (≥ 5 years). This distinction is important as patients might suffer from different issues during the treatment phase, then they do on the longer term (i.e. survivorship issues). First, some aspects of treatment toxicity only become apparent on the long-term, e.g. neurocognitive impairments caused by radiotherapy [2, 8–11]. Second, patients learn to adapt to the disease-related symptoms and change their coping strategies over time, influencing patients’ perception of their disease burden [2, 12]. Finally, on the long-term patients might face growth of tumor remnant or recurrence of disease, sometimes requiring intervention [13].

A methodological limitation of most published studies determining associations between certain risk factors and outcomes is the lack of distinction between determinants and predictors [14]. A determinant is an individual variable that on group-level is independently associated with the outcome of interest, corrected for confounding (e.g. the association between sex or tumor location with the long-term disease burden). Prediction models on the other hand use multiple variables together (i.e. patient, tumor and treatment characteristics) to predict for an individual patient the risk to develop a certain outcome of interest. Although both reflect patients’ future outcomes, determinants are variables with an assumed causal relationship to the outcome of interest (e.g. postoperative complications may have a negative impact on a patient’s long-term HRQoL), while predictors are solely used to predict the outcome of interest (e.g. hospitalization length may be predictive for diminished HRQoL on the long-term), without assuming causality.

We aimed to assess in meningioma patients determinants for the long-term disease burden, defined as impaired HRQoL and neurocognitive function at a median of 9 years after the last intervention. Furthermore, we have built prediction models to identify individual patients with a high risk around the time of intervention to suffer from a long-term impairment in HRQoL or neurocognitive function.

## Methods

### Participants

This is a secondary analysis of a multicenter cross-sectional study, assessing the long-term disease burden in meningioma patients [15]. Consecutive meningioma patients were recruited from the neurology, neurosurgery and radiation oncology outpatient clinics of two academic hospitals and one large non-academic teaching hospital between July 2016 and April 2019. Patients were eligible if the end of their anti-tumor treatment was at least 5 years prior to recruitment, or in case of active MRI surveillance, at least 5 years after diagnosis. Furthermore, patients had to be 18 years or older; with a histologically confirmed WHO grade I or II meningioma in case of surgery, and an MRI-based clinically suspected meningioma in case of radiotherapy only or active MRI surveillance. Exclusion criteria for study participation were history of whole brain radiotherapy, diagnosis with a neurodegenerative disease (including neurofibromatosis type II), or patients not proficient in the Dutch language.

### Procedures

Information on tumor and treatment was obtained from patient’s charts, and sociodemographic information was obtained at the beginning of the assessments (questionnaires and neurocognitive testing) from patients themselves. Radiological variables, such as tumor size and location, were assessed and recorded by the researchers to ensure uniformity of measurement. Clinician observed level of function was assessed using the Karnofsky Performance Score (KPS).

#### Patient-reported outcome measures

HRQoL was measured with the validated Short-Form Health Survey (SF-36), which yields 8 domain scores and two component scores [physical component summary (PCS) and mental component summary (MCS)], ranging from 0 to 100, with higher scores representing better HRQoL [16–18].

#### Neuropsychological assessment

Neuropsychological performance was assessed with a comprehensive battery of neuropsychological tests by trained research assistants and nurses: Digit-Symbol Substitution Test, Auditory Verbal Learning Test, Categoric Word Fluency Test, Concept Shifting Test, Memory Comparison Test, and Stroop Colour-Word Test [9, 10, 19]. Based on these tests, scores for the following neurocognitive domains were calculated: verbal memory, executive functioning, working memory, information processing speed, psychomotor functioning, and attention [9, 10, 19]. Each domain was transformed into z-scores, using means and standard deviations from a reference sample from the Maastricht Aging Study (MAAS; large longitudinal study on the psychological and biological determinants of cognitive aging), matched on group-level for age, sex and educational level [20].

### Statistical analysis

Multivariable regression analyses were performed to: (1) estimate the association between individual determinants, corrected for confounders, and impaired HRQoL and neurocognitive function, (2) build prediction models which could be used to predict the risk for impaired HRQoL or neurocognitive function for an individual patient based on patient-, tumor-, and treatment-related characteristics around diagnosis and intervention. Although for both analyses multivariable regression analyses are used, the statistical considerations and interpretation differ considerably. First, for the development of prediction models, only variables measured at baseline (around diagnosis and intervention) were included because the aim is to predict a future outcome. To assess determinants, variables later in the disease course were also considered (e.g. peritumoral edema before study assessment). Second, the outcomes of interest were dichotomized for the development of prediction models, as this facilitates use in clinical practice (i.e. does a patient have an impairment or not). For the analyses of determinants, outcomes were kept as continuous variables, as this increases statistical power.

Based on minimally clinically important differences as reported in the literature, HRQoL physical and mental component scores were dichotomized as follows: poor physical component score was defined as a score < 46.4 and poor mental component score as a score < 47.0 [21]. Impaired neurocognitive functioning was defined as a z-score < 1.5 in at least one out of six domains [22].

For all statistical tests, SPSS 23 (SPSS, Inc., Chicago, IL) was used, and p less than 0.05 was considered statistically significant.

#### Analysis of determinants

For the assessment of determinants, multivariable linear regression analyses were performed assessing the causal relationship between determinants (independent variable) and 5 outcomes (dependent variables): the SF-36 physical and mental component score (HRQoL), and z-scores for verbal memory, executive function, and attention (neurocognitive function). To reduce the number of analyses, only these 3/6 neurocognitive domains were chosen, as earlier analyses of this sample showed that patients primarily suffer from impairments in these domains [15]. Separate multivariable analyses were run for each association between a single determinant and a single outcome, corrected for possible confounders. A priori confounders were chosen for each analysis, based on prior knowledge and defined as associated with both the determinant and outcome, but not lying in the causal path between the determinant and outcome. Results were expressed as beta (β) with 95% confidence intervals, CIs [14].

#### Analysis of predictors

For prediction analyses we developed two multivariable logistic regression models for the following two dichotomous outcomes (dependent variables): impaired HRQoL (physical component score < 46.4 or mental component score < 47.0) and impaired neurocognitive function (z-score < 1.5 in at least one out of six domains). Based on the literature, clinically relevant variables were analyzed in univariable logistic regression analysis: gender, age, educational level, Charlson Comorbidity Index, tumor location and size, treatment characteristics (i.e. first resection, second resection, complications, radiotherapy), Simpson grading, WHO grade, years since diagnosis, and for the model predicting neurocognitive function also hand dominance. Variables were selected for multivariable analyses based on statistical significance in univariable regression analysis [3, 4, 7, 23–29]. A p < 0.20 as selection criterion was used to limit chances of overfitting. Sensitivity analyses were performed with a cut-off of p < 0.15. We assessed the discrimination for each model, using the area under the receiver-operating curve (AUC) including 95% confidence interval (CI). For each model we provided two patient examples showing how to calculate the absolute risk of impaired HRQoL or neurocognitive function for an individual patient.

## Results

A total of 190 patients (female: n = 149, 78%) were included in the analyses with a median follow-up since intervention of 9 years (IQR 7–12 years) (Table 1). Patients were on average 63 (SD 12) years old. Tumors were located on the skull base in 92 patients (48%), the cerebral convexity in 93 patients (49%) and other intracranial locations in 5 patients (3%). The majority of operated patients were classified with a WHO grade I meningioma (88%). Surgery was first line treatment in 168 (88%) patients, 36 (19%) received radiation.

**Table 1:** Sociodemographic and clinical characteristics of the included meningioma patients

	Meningioma patients n = 190
Age (years)	63 (SD 12)
Sex (female)	149 (78%)
Academic hospital (vs. nonacademic teaching hospital)	142 (75%)
Meningioma location	
Skull base	92 (48%)
Convexity	93 (49%)
Other	5 (3%)
Time since diagnosis (years)	10 (8–12)
Tumor size before intervention (mm)	38 (26–50)
Tumor size before study (mm)	0 (0–16)
Tumor growth on last MRI before study	10 (5%)
Number of meningiomas ≥ 2	26 (14%)
Active MRI surveillance	12 (6%)
Surgery as initial treatment	168 (88%)
Complication first surgery (operated patients: n = 168)	63 (38%)
Second surgery	13 (7%)
Time since first surgery (years)	9 (7–12)
Simpson grade (operated patients: n = 168)	
Grade I–III	109 (65%)
Grade IV–V	40 (24%)
Unknown	19 (11%)
WHO grade (operated patients: n = 168)	
Grade I	148 (88%)
Grade II	12 (7%)
Unknown	8 (5%)
Radiotherapy^a^	36 (19%)
Radiotherapy as initial treatment	10 (5%)
Adjuvant radiotherapy	26 (14%)
Time since radiotherapy (years)	8 (6–9)
Karnofsky Performance Status at time of study	100 (90–100)
Self-perceived neurocognitive impairment at time of study	94 (49%)
Self-reported motor dysfunction at time of study	55 (29%)
Dexamethasone use for symptoms at any moment during the care trajectory	22 (12%)
Physical rehabilitation	37 (19%)
Cognitive rehabilitation	8 (4%)
Psychological support	21 (11%)
Other supportive care	10 (5%)
Educational level	
Primary/secondary	40 (21%)
Tertiary: technical/vocational	85 (45%)
Academic	59 (31%)
Not provided	6 (3%)
Charlson Comorbidity Index	
0	127 (67%)
1≥	63 (23%)
Right handed	147 (77%)

^a^Radiotherapy techniques changed over time in each participating center, but all patients treated with radiotherapy received fractioned radiation

A total of 93 (49%) patients suffered from impaired HRQoL (PCS: n = 78, 41%; MCS n = 47, 53%), and 81 (43%) from objective neurocognitive deficits. A total of 127 (67%) suffered from a HRQoL impairment, *or* neurocognitive deficit.

### Determinants

#### HRQoL

Determinants for a lower physical component score (Table 2) were female sex (ref: male, β =  − 2.52, 95% CI − 6.39 to 1.35), increase in Charlson Comorbidity Index (β =  − 3.31 for each point increase, 95% CI − 4.62 to − 1.99), larger tumor size before study participation (β =  − 0.24, 95% CI − 0.45 to − 0.02), a lower educational level (β = 2.70, 95% CI 0.54 to 4.87), and lower KPS (β = 0.37, 95% CI 0.17 to 0.58). Determinant for a lower mental component score (Table 2) was lower KPS (β = 0.39, 95% CI 0.13 to 0.64). Tumor location, tumor size before intervention, surgical complications, reoperation, and radiotherapy were no determinants for HRQoL (Table 2).

**Table 2:** Determinants for Health-related Quality of Life as measured with the Short-from 36 (SF-36), separately for the physical and mental component score

	Physical component score β (95% CI)	Mental component score β (95% CI)
Sex female (ref: male)	** − 2.521 (− 6.393 to 1.351)**	0.066 (− 4.182 to 4.315)
Age (years)	− 0.113 (− 0.248 to 0.023)	− 0.016 (− 0.165 to 0.133)
Tumor location, skull base (ref: convexity)	2.832 (− 0.410 to 6.073)	2.603 (− 0.974 to 6.180)
Tumor size before last intervention (mm)	0.085 (− 0.017 to 0.187)	0.023 (− 0.086 to 0.132)
Tumor size before study (mm)	− **0.235 (**− **0.450 to** − **0.020)**	0.20 (− 0.211 to 0.252)
Tumor growth on last MRI before study, yes (no)	0.571 (− 1.479 to 2.626)	0.816 (− 1.396 to 3.029)
Edema on last MRI before study, yes (ref: no)	− 2.798 (− 10.988 to 5.392)	4.801 (− 4.077 to 13.678)
First resection, yes (ref: no)	1.438 (− 5.564 to 8.439)	3.072 (− 4.852 to 10.997)
First resection complications, yes (ref: no)	− 1.873 (− 5.596 to 1.851)	− 0.444 (− 4.648 to 3.760)
Second resection, yes (ref: no)	− 1.325 (− 8.290 to 5.640)	1.610 (− 6.590 to 9.811)
Simpson grade first resection IV/V (ref: I–III)	− 1.241 (− 3.001 to 0.519)	1.693 (− 0.216 to 3.602)
WHO Grade II (ref: I)	− 0.027 (− 6.657 to 6.603)	− 4.843 (− 11.988 to 2.301)
Radiotherapy, yes (ref: no)	− 2.950 (− 7.837 to 1.936)	− 3.327 (− 9.083 to 2.429)
Karnofsky performance score	**0.374 (0.170 to 0.578)**	**0.388 (0.133 to 0.643)**
Hand dominance, right (ref: left)	− 3.117 (− 7.694 to 1.460)	1.168 (− 3.815 to 6.152)
Charlson Comorbidity Index	− **3.308 (**− **4.624 to** − **1.992)**	− 0.021 (− 1.560 to 1.517)
Educational level (1: primary/secondary, 2: tertiary vocational, 3: academic)	**2.703 (0.540 to 4.867)**	0.762 (− 3.512 to 5.036)
Years since diagnosis	− 0.460 (− 0.500 to 0.410)	− 0.090 (0.720 to 0.400)

Results marked in bold are significant

β Represent the decrease or increase in physical or mental component score. For continuous determinants this is per 1-point increase in the determinant, unless otherwise specified. For categorical variables a comparison is made with a reference category

#### Neurocognitive function

Determinants for decreased neurocognitive function (Table 3) for all three selected domains were radiotherapy (range β − 1.06 to − 0.47), second resection (range β − 2.34 to − 0.62), higher age (range β − 0.05 to − 0.03), and lower educational level (range β for higher educational level: 0.31 to 0.91). Determinant for both decreased executive function and attention was lower KPS (range β 0.06 to 0.07). Determinants for worse executive function were maximum tumor size (β =  − 0.03 for each mm tumor, 95% CI − 0.05 to − 0.01) and edema on the last MRI before study participation (ref: no edema β =  − 0.84, 95% CI − 1.70 to − 0.01). Determinant for decreased attention was complications of first resection (β =  − 0.76, 95% CI − 1.42 to − 0.10). Tumor location, tumor size before intervention were no determinants for neurocognitive function (Table 3).

**Table 3:** Determinants for neurocognitive functioning as measured with a standardized test battery for the three previously determined most relevant domains in this patient population

	Verbal memory β (95% CI)	Executive function β (95% CI)	Attention β (95% CI)
Sex female (ref: male)	**0.442 (0.140 to 0.744)**	0.107 (− 0.332 to 0.546)	0.341 (− 0.350 to 1.032)
Age (years)	− **0.025 (**− **0.036 to** − **0.014)**	− **0.048 (**− **0.063 to** − **0.032)**	− **0.042 (**− **0.067 to** − **0.018)**
Tumor location, skull base (ref: convexity)	0.034 (− 0.223 to 0.290)	− 0.122 (− 0.499 to 0.255)	0.010 (− 0.588 to 0.608)
Tumor size before last intervention (mm)	− 0.004 (− 0.012 to 0.004)	− 0.005 (− 0.015 to 0.006)	− 0.004 (− 0.022 to 0.014)
Tumor size before study (mm)	− 0.007 (− 0.023 to 0.009)	− **0.028 (**− **0.051 to** − **0.005)**	− 0.024 (− 0.065 to 0.016)
Tumor growth on last MRI before study, yes (ref: no)	− 0.250 (− 1.336 to 0.836)	0.019 (− 0.194 to 0.231)	0.170 (− 0.181 to 0.521)
Edema on last MRI before study, yes (ref: no)	− 0.281 (− 0.892 to 0.330)	− **0.844 (**− **1.701 to** − **0.014)**	− 0.605 (− 2.023 to 0.813)
First resection, yes (ref: no)	**0.693 (0.130 to 1.256)**	− 0.069 (− 0.850 to 0.714)	− 0.279 (− 1.606 to 1.048)
First resection complications, yes (ref: no)	− 0.228 (− 0.553 to 0.097)	− 0.357 (− 0.761 to 0.047)	− **0.758 (**− **1.415 to** − **0.101)**
Second resection, yes (ref: no)	− **0.623 (**− **1.188 to** − **0.057)**	− **1.025 (**− **1.815 to** − **0.236)**	− **2.336 (**− **3.680 to** − **0.993)**
Simpson grade first resection IV/V (ref: I–III)	0.094 (− 0.040 to 0.229)	0.040 (− 0.147 to 0.227)	0.163 (− 0.167 to 0.492)
WHO Grade II (ref: I)	0.372 (− 0.104 to 0.847)	0.185 (− 0.530 to 0.899)	0.038 (− 1.108 to 1.184)
Radiotherapy, yes (ref: no)	− **0.469 (**− **0.866 to** − **0.071)**	− **0.666 (**− **1.224 to** − **0.107)**	− **1.063 (**− **2.036 to** − **0.090)**
Karnofsky performance score	0.012 (− 0.006 to 0.030)	**0.060 (0.035 to 0.085)**	**0.069 (0.025 to 0.113)**
Hand dominance, right (ref: left)	**0.520 (0.167 to 0.873)**	0.359 (− 0.168 to 0.886)	0.213 (− 0.693 to 1.065)
Charlson Comorbidity Index	− 0.019 (− 0.128 to 0.090)	− 0.125 (− 0.285 to 0.036)	− 0.133 (− 0.383 to 0.116)
Educational level (1: primary/secondary, 2: tertiary vocational, 3: academic)	**0.305 (0.139 to 0.471)**	**0.510 (0.265 to 0.756)**	**0.913 (0.528 to 1.297)**
Years since diagnosis	− 0.007 (− 0.045 to 0.031)	− 0.033 (− 0.090 to 0.023)	− 0.06 (− 0.140 to 0.030)

Results marked in bold are significant

β Represent the decrease or increase in z-score. For continuous determinants this is per 1-point increase in the determinant, unless otherwise specified. For categorical variables a comparison is made with a reference category

### Prediction models

#### HRQoL impairments

Using a p-value cut-off < 0.20 in univariable analyses, the following variables were included in the multivariable prediction model: age, tumor size before intervention, surgery, surgical complications, Charlson Comorbidity Index and educational level (Table 4). This model showed an AUC of 0.72 (95% CI 0.63 to 0.80) (Supplementary Fig. 1). Sensitivity analysis resulted in a model with the same variables, except for age, with also a similar AUC of 0.72. The full prediction model to calculate the absolute risk of impaired HRQoL is presented in Supplementary Table 1.

**Table 4:** Prediction model development for impaired Health-related quality of life

	Univariable analysis odds ratio (95% CI)	Multivariable model based on statistical significance only odds ratio (95% CI)
Sex female (ref: male)	1.024 (0.505 to 2.076), p = 0.947	
Age (years)	1.018 (0.992 to 1.044), p = 0.173	0.997 (0.964 to 1.030)
Tumor location, skull base (ref: convexity)	0.801 (0.446 to 1.437), p = 0.456	
Tumor size before last intervention (mm)	0.982 (0.964 to 1.001), p = 0.061	0.980 (0.959 to 1.002)
First resection yes (ref: no)	0.408 (0.158 to 1.052), p = 0.064	0.438 (0.117 to 1.637)
First resection complications yes (ref: no)	2.066 (1.102 to 3.873), p = 0.024	1.924 (0.900 to 4.114)
Second resection yes (ref: no)	1.406 (0.411 to 4.804), p = 0.587	
Simpson grade first resection IV/V (ref: I–III)	1.502 (0.724 to 3.118), p = 0.275	
WHO Grade II (ref: I)	1.772 (0.537 to 5.845), p = 0.348	
Radiotherapy yes (ref: no)	1.610 (0.575 to 3.421), p = 0.216	
Charlson Comorbidity Index	1.520 (1.117 to 2.069), p = 0.008	1.338 (0.930 to 1.925)
Educational level (1: primary/secondary, 2: tertiary vocational, 3: academic)	0.535 (0.351 to 0.816), p = 0.004	0.428 (0.255 to 0.717)
Years since diagnosis	1.036 (0.953 to 1.127), p = 0.406	

Health-related quality of life impairment is defined as a physical component score < 46.4 or mental component score < 47.0). p-values are only showed for the univariable analysis, as they were used for development of the multivariable model that was based on statistical significance

#### Neurocognitive impairments

Using a p-value cut-off < 0.20 in univariable analyses, the following variables were included in the multivariable prediction model: age, tumor size before intervention, reresection, radiotherapy, educational level, and years since diagnosis (Table 5). This model showed an AUC of 0.78 (95% CI 0.70 to 0.85) (Supplementary Fig. 1). Sensitivity analysis resulted in the same model with the same variables and hence the same AUC. The full prediction model to calculate the absolute risk of impaired neurocognitive function is presented in Supplementary Table 2.

**Table 5:** Prediction model development for Neurocognitive deficits

	Univariable analysis odds ratios (95% CI)	Multivariable model based on statistical significance odds ratios (95% CI)
Gender female (ref: male)	1.089 (0.540 to 2.196), p = 0.813	
Age (years)	1.036 (1.008 to 1.064), p = 0.011	1.024 (0.987 to 1.063)
Tumor location, skull base (ref: convexity)	1.072 (0.598 to 1.923), p = 0.816	
Tumor size before last intervention (mm)	1.019 (1.000 to 1.039), p = .048	1.022 (0.998 to 1.047)
First resection yes (ref: no)	0.729 (0.299 to 1.777), p = 0.487	
First resection complications yes (ref: no)	1.500 (0.805 to 2.794), p = 0.201	
Second resection yes (ref: no)	4.574 (1.191 to 17.572), p = 0.027	2.662 (0.488 to 14.528)
Simpson grade first resection IV/V (ref: I–III)	1.121 (0.540 to 2.325), p = 0.760	
WHO Grade II (ref: I)	2.148 (0.651 to 7.092), p = 0.210	
Radiotherapy yes (ref: no)	2.011 (0.956 to 4.230), p = 0.066	2.819 (0.925 to 8.585)
Hand dominance, right (ref: left)	0.659 (0.289 to 1.505), p = 0.323	
Charlson Comorbidity Index	1.135 (0.877 to 1.468), p = 0.336	
Educational level (1: primary/secondary, 2: tertiary vocational, 3: academic)	0.412 (0.265 to 0.641), p = 0.000	0.359 (0.206 to 0.628)
Years since diagnosis	1.103 (1.011 to 1.203), p = 0.027	1.130 (0.982 to 1.301)

Neurocognitive deficit is defined as a z-score < 1.5 in at least one neurocognitive domain. p-values are only showed for the univariable analysis, as they were used for development of the multivariable models that was based on statistical significance

#### Examples

Example patients and calculations are provided for both prediction models in Supplementary Tables 1 and 2. Furthermore, using the predicted risk for HRQoL impairment, the sample was divided into tertiles (i.e. three equally large groups: low-risk, medium-risk, high-risk). Of the patients in the low-risk group 27% suffered an HRQoL impairment, 40% in the medium-risk group, and 70% in the high-risk group. Using the predicted risk for neurocognitive impairment to divide patients in risk groups, 9% of patients in the low-risk group suffered from a neurocognitive impairment, 47% in the medium-risk group, and 60% of patients in the high-risk group.

## Discussion

Results of this study indicate that determinants for the long-term disease burden in meningioma patients on group level are (1) sociodemographic characteristics: sex, age and educational level, (2) treatment characteristics: complications of surgery, reoperation, radiotherapy, (3) tumor characteristics: diameter and peritumoral edema at the time of study, and (4) clinician-reported level of functioning (i.e. KPS). Furthermore, we have developed prediction models to predict whether an individual patient will suffer from long-term HRQoL or neurocognitive impairment using easily accessible patient chart information, which showed moderate to good discriminative ability to differentiate between those with and without clinically relevant impairments in HRQoL or neurocognitive function on the long term. We reported that 67% of patients suffered from impaired HRQoL or neurocognitive deficits. For these patients, rehabilitation and supportive care options should be available, even on the long-term, as the need for these supportive treatments was underlined in a previous study in meningioma patients [30]. In this study we focused on readily available variables as determinants and predictors, facilitating use in daily clinical practice.

### Interpretation: meningioma literature on determinants for disease burden

Information on determinants might be useful for clinicians to better understand the impact of both the tumor and treatment on the long-term outcomes of patients. We report that a complicated treatment course with surgical complications, the need for reoperation and radiotherapy, are associated with long-term neurocognitive impairments and less with HRQoL impairments, which is in line with the literature on (low grade) glioma patients [9, 31]. On a group-level, meningioma patients therefore deserve extra attention regarding neurocognitive deficits and early referral for neurocognitive rehabilitation. Furthermore, results of this study showed that tumor activity at the time of study, defined as the presence of edema and a larger tumor diameter on the last MRI before study participation, were negatively associated with patients’ executive function. A larger tumor diameter was also associated with decreased physical function. This is in line with previous meningioma studies reporting in the first years after treatment that factors negatively influencing overall HRQoL and neurocognitive function were higher histological grade, a larger tumor size and peritumoral edema [23, 25, 32]. However, we found no association between WHO grade and HRQoL or neurocognitive function, which might be explained by the low number of patients with WHO grade II tumors in our study (7%). Indeed, based on the WHO 2016 classification of central nervous system tumors, WHO grade II tumors occur in up to 20% of patients [1]. Our results may therefore not be completely generalizable, as we have a slight underrepresentation of patients with WHO grade II tumors. Two previous studies reported, using univariable analyses only, that tumor location and tumor laterality were associated with neurocognitive function, while in the current study no association was observed after correction for confounders [7, 25, 27, 28]. These results have implications for our understanding of the disease burden in meningioma, as generally it is thought that patients with skull base lesions, compared with convexity tumors, suffer from worse HRQoL after surgery [3].

### Interpretation: prediction models for individual meningioma patients

Prediction models were developed to estimate which patient develops a long-term impairment in HRQoL or neurocognitive function. Until now there have been no prediction models developed for the short- or long-term disease burden of meningioma patients. Not only does the disease burden changes over time, as HRQoL and neurocognitive impairments become more prominent after 5 years of follow-up [3–5, 15, 29]. It has also been acknowledged that patients enter a chronic disease state in the long-term, with specific long-term survivor issues [3, 4]. With good survival rates of this patient population, a prediction model for the long-term disease burden is of particular interest. Two separate models were built, one for long-term problems in HRQoL and one for neurocognitive impairments. These models showed that higher age, lower educational level, presence of comorbidities as measured with the Charlson Comorbidity Index, larger tumor size before intervention, surgical complications, the need for reresection, initiation of radiotherapy, and years since diagnosis were predictors for long-term impairments. Although these variables together help to predict these future outcomes, not all of these variables were independently related to the measured outcomes (i.e. determinants), such as tumor location. This emphasizes the difference between predictors and determinants. While determinants are variables causally related to the outcome of interest, predictors are solely used to predict the outcome of interest, without assuming causality. Hence, predictors can be determinants, act as a proxy for a determinant, or have no causal relationship at all with the long-term disease burden.

### Limitations

The measured outcomes in this study are 9 years after the last intervention. Therefore, the studied patients might have experienced other major health issues and undergone large extracranial treatments between the period of meningioma treatment and study participation, which could impact their long-term HRQoL and neurocognitive function. Furthermore, a limitation of the current study is the lack of external validation of the models. Prediction models that are only internally validated might be overfitted with externally validated models showing lower performance measures. This might especially hold true for the models predicting HRQoL, as it is strongly subjected to the sociocultural context and different health care systems. Cross-cultural validation is therefore warranted. Furthermore, due to the cross-sectional nature of our study, we were unable to assess determinants and predictors for a change in HRQoL or neurocognitive function over time. Previous studies have shown that baseline HRQoL also acts as predictor for long-term HRQoL, which is a more precise measure of functioning than the KPS [3]. In the light of lack of a validated meningioma-specific HRQoL instrument, we used the SF-36 to measure HRQoL, as this is the most frequently used HRQoL instrument in meningioma literature and in other diseases, facilitating comparability of our results [3]. However, HRQoL issues specific to this patient group might therefore be missing [2]. Previous research has indeed shown that existing HRQoL questionnaires currently used in meningioma patients do not fully cover all relevant issues, supporting the need to develop and validate a meningioma-specific HRQoL questionnaire.

### Implications for clinical practice

The found determinants can help clinicians to better understand the long-term HRQoL and neurocognitive impairments of patients, as both the impact of the tumor and the treatment they initiate may affect patients’ functioning and well-being. The prediction models can be used to identify individual patients at baseline with a high risk to suffer from a long-term disease burden, which enables tailored provision of information and allocation of scarce supportive care services to those most likely to obtain benefit. Our results emphasize that predictors are not per se determinants, and that causal attributions shouldn’t be given to predictors. We recommend external validation in the country of the population of interest before clinical use of the described prediction models.

## Electronic supplementary material

Below is the link to the electronic supplementary material.

Supplementary file1 (PDF 356 kb)

## Data Availability

Upon request (please direct to amir@lumc.nl) the used code for the analysis can be provided.

## Code availability

Upon request (please direct to amir@lumc.nl) the used code for the analysis can be provided.
