# Supplementary material for: Determinants and predictors for the long-term disease burden of intracranial meningioma patients
Source: J Neurooncol. 2020 Oct 19;151(2):201–10. doi: 10.1007/s11060-020-03650-1 (PMC7875939; doi:10.1007/s11060-020-03650-1)
Supplement: Supplementary file 1 — Supplementary file1 (PDF 356 kb) [file 11060_2020_3650_MOESM1_ESM.pdf]

Supplementary Figure 1: Receiver Operating Curves (ROC) for the developed multivariable models and Area Under the ROC (AUC)

Model Health-related quality of life: AUC 0.717 (95%CI 0.633 to 0.801)

Model Neurocognitive deficits: AUC 0.775 (95%CI 0.696 to 0.853)

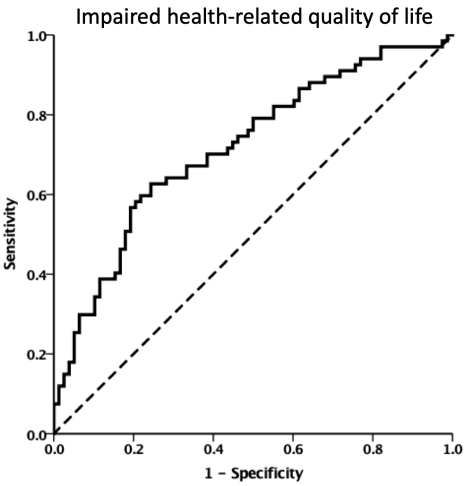

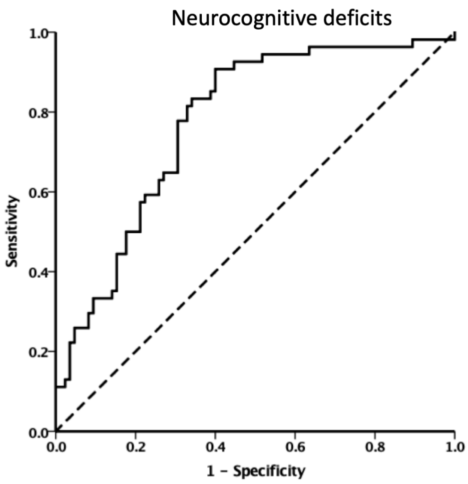

Supplementary Table 1: Health-related Quality of Life (HRQoL)

**Formula for full risk score:**	y=2.997 + (-0.003x age in years) + (-0.020 x largest tumor diameter before first intervention) + (-0.826 x surgery[yes]) + (0.655 x surgical complications[yes]) + (0.291 x Charlson Comorbidity Index) + (-0.849 x education level)
**Explanation:**	2.997 is the intercept of the model. Largest tumor diameter before first intervention was measured in mm. Charlson comordidity index ranges from 0 to 30). Education is classified as (1=primary/secondary, 2=tertiary vocational, 3 academic).
**Formula for impaired HRQoL**	HRQoL= 1/1+e^{-y}
**Example 1:**	80 years old patients with a skull base tumor of a maximum diameter of 44 millimetre who received surgery, with surgical complications, with a Charlson Comorbidity Index of 6, who only followed primary education: y=2.997 + (-0.003 x 80) + (-0.020 x 44) + (-0.826 x 1) + (0.655 x 1) + (0.291 x 6) + (-0.849 x 1) =2.603<br>Chance for impaired HRQoL = 1/1+$e^{-2.603}$=93%
**Example 2:**	40 years old patients with a skull base tumor of a maximum diameter of 11 millimetre who received only surgery, without a surgical complications, with a Charlson Comorbidity Index of 2, who followed academic education: y=2.997 + (-0.003 x 40) + (-0.020 x 11) + (-0.826 x 1) + (0.655 x 0) + (0.291 x 2) + (-0.849 x 3) =-0.134<br>Chance for impaired HRQoL = 1/1+$e^{0.134}$=47%

Supplementary Table 2: Neurocognitive function

**Formula for full risk score:**	y=-2.212 + (0.024x age in years) + (0.022 x largest tumor diameter before first intervention) + (0.979x reresection[yes]) + (1.036 x radiotherapy[yes]) + (-1.023 x education level) + (0.123 x years since diagnosis).
**Explanation:**	2.212 is the intercept of the model. Largest tumor diameter before first intervention was measured in mm. Education is classified as (1=primary/secondary, 2=tertiary vocational, 3 academic).
**Formula for impaired neurocognitive function:**	Impaired neurocognitive function = $1/1+e^{-y}$
**Example 1:**	80 years old patient with a maximum tumor diameter of 44 millimetre who was operated twice and received radiotherapy, who only followed primary education, 9 years after diagnosis: y=-2.212 + (0.024x80) + (0.022 x 44) + (0.979 x 1) + (1.036 x 1) + (-1.023 x 1) + (0.123 x 9)=2.775.<br>Chance for impaired neurocognitive function = $1/1+e^{-2.775}$=94%
**Example 2:**	40 years old patients with a maximum tumor diameter of 11 millimetre who was operated twice and who followed academic education, 9 years after diagnosis: y=-2.212 + (0.024x40) + (0.022 x 11) + (0.979 x 1) + (1.036 x 0) + (-1.023 x 3) + (0.123 x 9)=-1.993.<br>Chance for impaired neurocognitive function = $1/1+e^{1.993}$ =12%
